# Red and Green Fluorescence from Oral Biofilms

**DOI:** 10.1371/journal.pone.0168428

**Published:** 2016-12-20

**Authors:** Catherine M. C. Volgenant, Michel A. Hoogenkamp, Bastiaan P. Krom, Marleen M. Janus, Jacob M. ten Cate, Johannes J. de Soet, Wim Crielaard, Monique H. van der Veen

**Affiliations:** 1 Department of Preventive Dentistry, Academic Centre for Dentistry Amsterdam (ACTA), University of Amsterdam and Vrije Universiteit Amsterdam, Amsterdam, the Netherlands; 2 Top Institute Food and Nutrition, Wageningen, the Netherlands; Oregon Health & Science University, UNITED STATES

## Abstract

Red and green autofluorescence have been observed from dental plaque after excitation by blue light. It has been suggested that this red fluorescence is related to caries and the cariogenic potential of dental plaque. Recently, it was suggested that red fluorescence may be related to gingivitis. Little is known about green fluorescence from biofilms. Therefore, we assessed the dynamics of red and green fluorescence in real-time during biofilm formation. In addition, the fluorescence patterns of biofilm formed from saliva of eight different donors are described under simulated gingivitis and caries conditions. Biofilm formation was analysed for 12 hours under flow conditions in a microfluidic BioFlux flow system with high performance microscopy using a camera to allow live cell imaging. For fluorescence images dedicated excitation and emission filters were used. Both green and red fluorescence were linearly related with the total biomass of the biofilms. All biofilms displayed to some extent green and red fluorescence, with higher red and green fluorescence intensities from biofilms grown in the presence of serum (gingivitis simulation) as compared to the sucrose grown biofilms (cariogenic simulation). Remarkably, cocci with long chain lengths, presumably streptococci, were observed in the biofilms. Green and red fluorescence were not found homogeneously distributed within the biofilms: highly fluorescent spots (both green and red) were visible throughout the biomass. An increase in red fluorescence from the *in vitro* biofilms appeared to be related to the clinical inflammatory response of the respective saliva donors, which was previously assessed during an *in vivo* period of performing no-oral hygiene. The BioFlux model proved to be a reliable model to assess biofilm fluorescence. With this model, a prediction can be made whether a patient will be prone to the development of gingivitis or caries.

## Introduction

Quantitative light induced fluorescence (QLF) is gaining recognition as an established method for examining the oral cavity in clinical dental research. This technique uses the auto-fluorescence characteristics of teeth at excitation wavelength 405 nm to detect possible mineral loss from enamel surfaces which is related to fluorescence loss of a tooth [1, 2]. This mineral loss is the clinical starting point of dental caries in teeth. Besides this useful application of the QLF technique, red fluorescence is observed with QLF on surfaces where dental plaque or calculus is present [3–5], although not all dental plaque on teeth is fluorescing red [6].

The long-term presence of a pathogenic biofilm (dental plaque) on the teeth is the main cause of oral infectious diseases such as dental caries and gingivitis [7]. The composition of plaque associated with health is different compared to pathogenic plaque [8, 9]. A local dysbiosis of the plaque is characteristic for periodontitis (severe inflammation of the supportive tissue of teeth [10]), as well as for caries [11]. Consequently, the visualisation and elimination of dysbiotic plaque may be a key preventive method to know in who and where to restore the balance in the biofilm to prevent further development of disease.

Previous research has suggested that red plaque fluorescence is associated with dental plaque cariogenicity [12–15]. In addition, a recent clinical study reported that, within a period of 14 days without oral hygiene, the presence of red fluorescence in 2 days old plaque is a predictive marker for the inflammatory response of the gingiva at day 14 [16]. The inflammation of the gingiva in this study was determined by the bleeding on marginal probing index, as described by Van der Weijden *et al*. [17]. Remarkably, this clinical research revealed big differences among the participants: some developed a considerable amount of red fluorescent dental plaque, where others did have dental plaque, but no red fluorescence. This low-fluorescence group had also less gingival inflammation after 14 days without oral hygiene. Moreover, this difference in red fluorescence was already visible after 24 hours, although not statistically significant.

A landmark study in dentistry has reported that bleeding on probing increases when plaque remains present during a longer period of refraining from oral hygiene [18]. Therefore bleeding on (marginal) probing is often considered as an indication of the average level of oral hygiene and gingival inflammation. This is a proper method to check the current situation in the mouth, but it does not give the dentist information about the resilience of the mouth of a patient: its ability to recover quickly from a sudden change in the local environment. An example of a change in the oral environment is an increase in the frequency of the carbohydrates intake in the diet of a patient. To prevent future oral diseases, professional dental care should focus on the early signs of dental plaque dysbiosis.

Because of the presumed relation with caries and gingivitis, the presence or absence of red fluorescence may give an indication of oral health. However, little is known about the characteristics of red fluorescence from oral biofilms, the influence of external factors on its fluorescence (like nutrition and oral rinses) on this biofilm fluorescence and the interpretation of green biofilm fluorescence. An explanation for the red fluorescence from dental plaque may be found in the molecules involved in gingivitis and periodontitis: gingival inflammation leads to the production of gingival crevicular fluid, which contains relative high concentrations of heme [19]. Heme is required as a source of iron by oral bacteria like *Porphyromonas gingivalis* for their growth [20]. The heme molecule consists of a protoporphyrin IX ring with a central ferrous atom. Protoporphyrin IX is suggested to be the source of the red fluorescence in the dental plaque [12], which could explain plaque red fluorescence when the gingival tissues are inflamed. In this study we examined the real-time dynamics of red and green fluorescence during biofilm formation. Furthermore, we described the patterns of fluorescence of biofilm formed from saliva of different donors. An increased red fluorescence of the biofilms appeared to be related to the clinical inflammatory response of the respective saliva donors.

## Materials and Methods

### Saliva collection

The saliva samples used to inoculate the biofilm system in this study were derived from participants of a previous cohort study [16]. This clinical study was conducted in accordance with the ethical principles of the 64th WMA Declaration of Helsinki (October 2013, Brazil) and the Medical Research Involving Human Subjects Act (WMO), approximating Good Clinical Practice (CPMP/ICH/135/95) guidelines. Approval for this study was obtained by the Medical Ethical Committee of the VU Medical Center (2014.505). At the screening session, volunteers received oral and written information about the study and all participants of the clinical study signed the informed consent. Inclusion and exclusion criteria are described elsewhere [16].

Baseline saliva samples from eight systemically and orally healthy donors from this study were selected and used for the present study. Out of the eight samples, four saliva samples were from low responders and four from high responders regarding red fluorescent plaque build-up during the experimental period [16]. The participants did not perform any oral hygiene measures for 24 hours nor did they drink or eat in the two hours prior to saliva donation. Stimulated saliva was collected and stored as described by Janus *et al*. 2015 [21].

### Salivary microcosm biofilm growth

**Fig 1:**
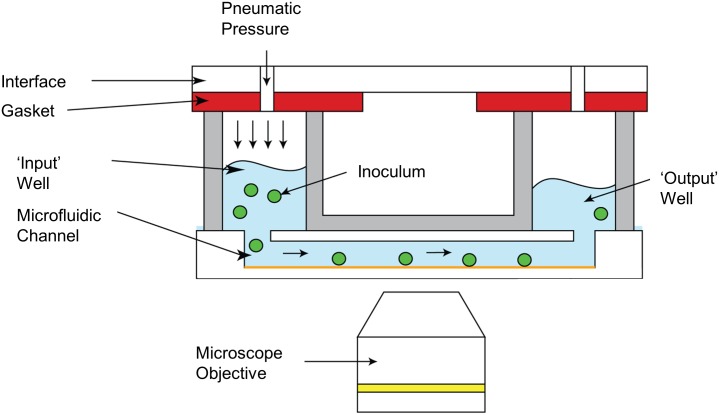
Overview of the BioFlux biofilm model. Images courtesy of Fluxion Biosciences Inc., South San Francisco, CA, USA

Biofilm formation was analysed under flow conditions in the microfluidic BioFlux 1000z (Fluxion Biosciences Inc., South San Francisco, CA, USA). This system was developed for automated, high throughput shear flow assays and is used in combination with high performance microscopy (Fig 1, Axio Observer Z1, Zeiss, Jena, Germany), equipped with a black and white CCD-camera (Exi Aqua Bio, QImaging, Surrey, Canada) to allow for live cell imaging. All liquids used in this experiment were pre-warmed to 37°C, to prevent gas bubble formation within the viewing plane. Prior to inoculation, the channels of a Fluxion, 48-well plate [22] were primed with 200μL phosphate buffered saline to fill the channels and to remove air from the system.

To obtain salivary microcosm biofilms, previously collected saliva samples were thawed and sonicated on ice, 30 times, 1 s, at 40 Hz (VC130 Ultrasonic processor, Sonics & Materials Inc., Newtown, CT, USA). Subsequently, 75 μL of saliva was added to the outlet well of a separate channel and back-flowed at 0.1 dyne until the channel was filled. The plate was incubated for 1 h at 37°C to allow for microbial attachment. To stimulate biofilm development 1.5 mL modified artificial saliva medium [23] supplemented with either 10% (v/v) fetal bovine serum (Sigma-Aldrich, St. Louis, MO, USA) or 0.2% (v/v) sucrose (Merck, Darmstadt, Germany) was added to the inlet wells of each channel and flowed through the channels at 0.5 dyn/cm^2^ for 12 h at 37°C under anaerobic conditions (80% N_2_, 10% H_2_, 10% CO_2_). Additionally the sucrose fed microcosms were also grown under aerobic conditions to determine the effect of aerobiosis on microcosm growth and fluorescence.

To assess the development of microcosm biofilm and the red and green fluorescence developing in time, three regions of interest (ROI) per channel were randomly picked resulting in six ROIs per experiment. The dimensions of a channel were 75x350 μm. The width of the channel corresponded with the height of an image. Real-time fluorescence and brightfield images (20x objective, see Table 1 for light and filter settings) were acquired every 10 min for 12 hour using the BioFlux Meta Imaging Series Software Version 7.8.1 (Molecular Devices, Downingtown, PA, USA). For the fluorescence images dedicated excitation and emission filters were used (Table 1, Chroma Technology Corporation, Bellows Falls, VT, USA). Each saliva sample was grown twice in duplicate for biofilm growth for all three conditions. This resulted in four individual studied biofilms per saliva sample per growth condition.

**Table 1:** Filter block characteristics.

Light signal	λ_ex/bandwidth [nm]_	λ_em/bandwidth [nm]_	Dichroic filter	Exposure time
**Green**	405/30	520/40	T495plxt	150 ms
**Red**	405/30	630/75	T495plxt	250 ms
**Brightfield**	-	-	-	10 ms

### Image analysis

After image acquisition stacks were built and the images were analysed for anomalies (e.g., trapped air), stacks with trapped air were subsequently removed before further analysis. Of each ROI, total intensity for the brightfield, green fluorescence and red fluorescence images in a stack was calculated and plotted using the Time Series Analyser plugin Version 3.0 (Balaji J, Dept. of Neurobiology, UCLA, Los Angeles, CA, USA) of the ImageJ software Version 1.50g (rsb.info.nih.gov/ij).

A moving average of 10 time points was calculated for each ROI to correct for fluctuations in total intensity due to small clumps of biofilm floating through the channels. Subsequently, the average corrected total intensity per growth condition (2 channels, 3 ROIs, 3 experiments *in duplo*) was calculated per time point. The inverted brightfield intensity was used as measure of total biomass. The average intensities of green biofilm fluorescence and red biofilm fluorescence are considered a measure green and red fluorescent biomass, respectively. These three assessments were subsequently transformed into a percentage relative to the first (averaged) time point. All obtained data is included in S1 Table. Raw images were used to assess intensities after which brightness and contrast were adjusted in ImageJ to create comprehensible time-lapse ‘movies’ (See S1–S6 Movies: with green respectively red fluorescence as composite overlaying the brightfield biofilm images).

## Results

### Biofilm growth

**Fig 2:**
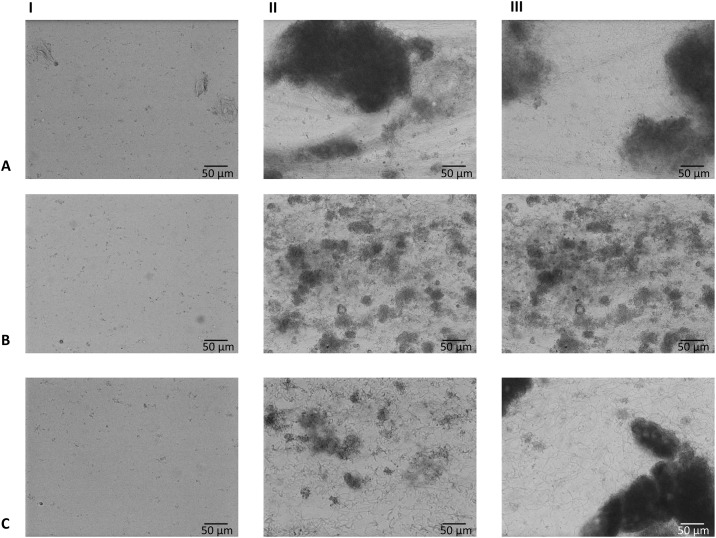
Brightfield images showing typical biofilms. This example was cultured from inoculum from a clinically low-fluorescent, low-bleeding on marginal probing participant. **A:** anaerobic biofilm grown with artificial saliva medium and 10% serum; **B:** anaerobic biofilm grown with artificial saliva medium and 0.2% sucrose; **C:** aerobic biofilm grown with artificial saliva medium and 0.2% sucrose. **I:** at time point 0 (one hour after inoculation); **II:** after 6 hours of biofilm growth; **III:** after 12 hours of biofilm growth.

Image analysis showed air entrapment in 10 out of 96 channels, which were excluded from analysis. This phenomenon was evenly distributed over the different saliva samples and the three growth conditions. Typical examples of biofilm formation and biofilm fluorescence are given in Fig 2. Typical examples of time-lapse movies of biofilm formation with green and red fluorescence are included as supplementary material (S1–S6 Movies).

**Fig 3:**
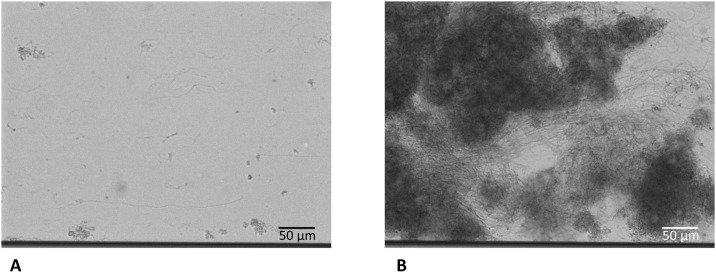
Typical example of the long chains as observed from all three growth conditions. This example is from a biofilm which was grown with artificial saliva medium and 10% serum (anaerobe) (**A**) after 4 hours of biofilm growth and (**B**) after 6 hours of biofilm growth. This example was cultured from inoculum from a clinically high-fluorescent, high-bleeding on marginal probing participant. The dark line on the bottom of the image is the border of the channel.

Small differences were seen from the biofilms of the three conditions (artificial saliva medium with serum (anaerobically) or sucrose (anaerobically and aerobically)). Total biomass, represented as an increase of the average intensity of the inverted brightfield signal, was lower in the anaerobic sucrose group (S1 Fig). Clumps of thick masses of biofilm were present in all biofilms. In all biofilms, long chains of presumably streptococci were observed (typical example shown in Fig 3. A summary of the results is given in Table 2 (full results in S1 Fig), together with the clinical characteristics of all eight donors.

**Table 2:** Overview of *in-vitro* red fluorescence as observed in medium with serum.

Saliva number	Gender of the donor	Age of the donor	Clinical percentage of red plaque fluorescence after a 14 days no oral hygiene challenge [16]	BOMP (%) after a 14 days no oral hygiene challenge [16]	*In-vitro* red fluorescence, anaerobe serum group	*In-vitro* red fluorescence, both sucrose groups
**1**	Male	23	23	53	High	Low
**2**	Female	30	21	37	High	Low
**3**	Male	23	27	42	High	Low
**4**	Female	24	3	45	High	Low
**5**	Female	26	21	10	Low	Low
**6**	Female	20	0	5	Low	Low
**7**	Female	19	0	8	Low	Low
**8**	Female	29	0	2	Low	Low

The clinical percentage of red plaque fluorescence refers to the amount of red fluorescent dental plaque present after 14 days no oral hygiene [16]. The BOMP refers to the percentage of sites in the mouth which are bleeding on marginal probing after 14 days no oral hygiene [16].

### Fluorescence microscopy

The BioFlux microfluidic system proved to be a reproducible model for detection and quantification of green and red fluorescence from microcosm biofilms. No signs of photo bleaching of fluorescence was observed.

**Fig 4:**
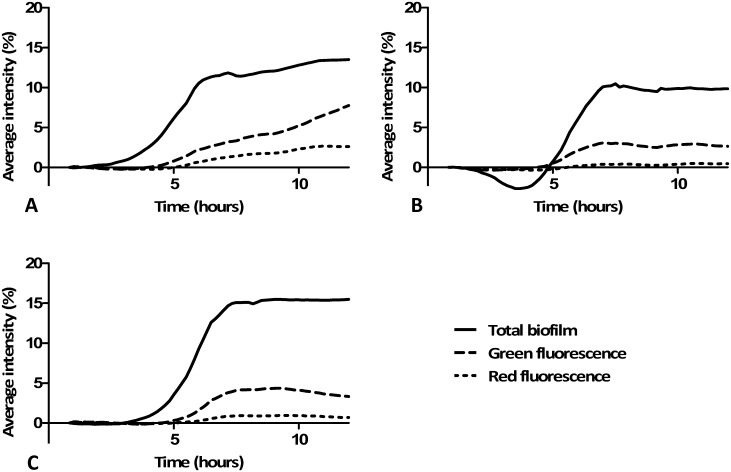
Average biofilm growth with its fluorescence. The graphs show a typical example of biofilm growth with its green and red fluorescence. **A:** anaerobic biofilm grown with artificial saliva medium and 10% serum; **B:** anaerobic biofilm grown with artificial saliva medium and 0.2% sucrose; **C:** aerobic biofilm grown with artificial saliva medium and 0.2% sucrose. The reduction of total biofilm in (**B**) can be explained by clumps of buccal cells, present at inoculation, which are at later flowed away due to the continuous medium flow in the model. This example was grown from inoculum from a clinically high-fluorescent, high-bleeding on marginal probing participant. Results from the other seven inocula are shown in S1 Fig.

**Fig 5:**
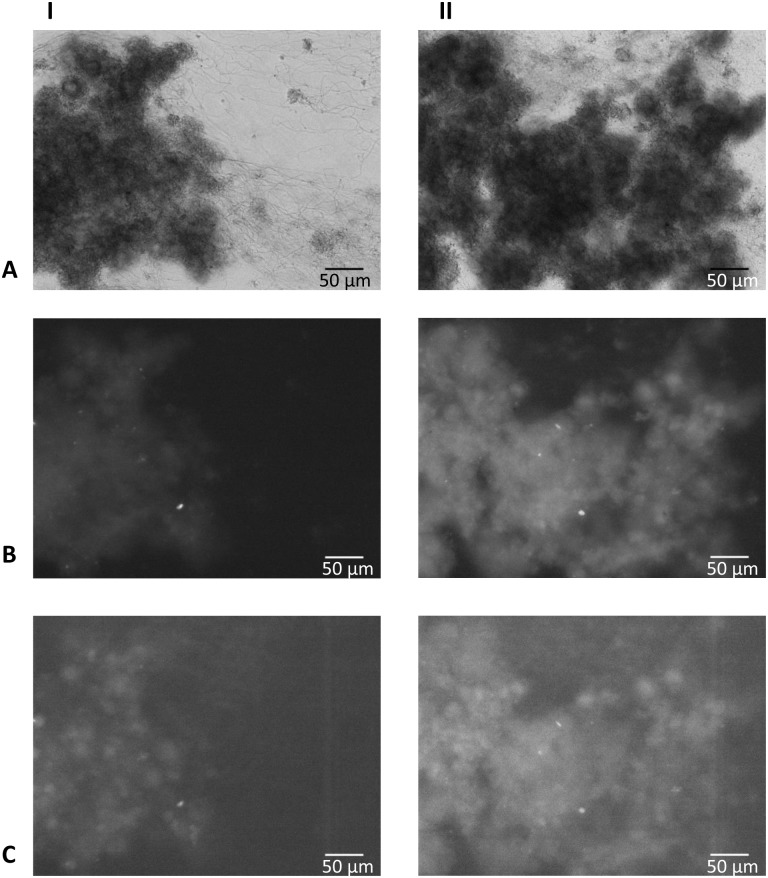
Brightfield and fluorescence images showing typical biofilms from the serum group. These biofilms were grown from inoculum of a clinically high-fluorescent, high-bleeding on marginal probing participant. **A:** brightfield image; **B:** fluorescence image obtained using a green fluorescence filter; **C:** fluorescence image obtained using a red fluorescence filter; **I:** after 6 hours of biofilm growth; **II:** after 12 hours of biofilm growth.

Green and red fluorescence were observed from all three conditions and from all eight inocula. Green and red fluorescence intensities were in line with the total biomass present, with red fluorescence having a much lower intensity compared to the green fluorescence. The highest intensity of green fluorescence was measured in the (anaerobic) serum group (Fig 4A and S1A Fig). The highest relative intensity of red fluorescence was also observed in the (anaerobic) serum group, but only for four out of eight biofilm groups, which was highly correlated to *in vivo* gingival inflammation of the donors as shown in Table 2. Green and red fluorescence were found early in biofilm formation (Fig 5). Green and red fluorescence were not homogeneously distributed within the biofilms (Fig 5) as clear high fluorescent spots (red and green) were visible throughout the biofilm.

Correlation coefficients between the different biofilm signals (total biomass, green and red fluorescence) are presented in Table 3: all correlations were strong and significant, except for the anaerobic groups (serum and sucrose) of saliva from donor six. These biofilms showed little fluorescence, explaining the absence of a correlation (S1 Fig). More red fluorescence was observed from anaerobic biofilms (S1 Fig), but this was in line with the amount of biomass (Table 3).

**Table 3:** Correlation coefficients between different biofilm signals.

Growth condition		Serum (anaerobe)		Sucrose (anaerobe)		Sucrose (aerobe)	
	Comparison	Correlation coefficient r_s_	B (95% CI)	Correlation coefficient r_s_	B (95% CI)	Correlation coefficient r_s_	B (95% CI)
**Saliva 1**	TB—GF	0.755**	0.218 (0.183–0.253)	0.905**	0.249 (0.239–0.260)	0.920**	0.259 (0.244–0.273)
	TB—RF	0.574**	0.110 (0.084–0.137)	0.744**	0.052 (0.047–0.057)	0.898**	0.068 (0.063–0.074)
	GF—RF	0.881**	0.569 (0.534–0.605)	0.863**	0.211 (0.196–0.225)	0.989**	0.268 (0.260–0.276)
**Saliva 2**	TB—GF	0.957**	0.435 (0.386–0.484)	0.936**	0.264 (0.255–0.273)	0.850**	0.264 (0.253–0.278)
	TB—RF	0.900**	0.183 (0.162–0.204)	0.910**	0.056 (0.052–0.059)	0.807**	0.059 (0.055–0.063)
	GF—RF	0.954**	0.418 (0.403–0.434)	0.867**	0.204 (0.188–0.221)	0.972**	0.224 (0.219–0.230)
**Saliva 3**	TB—GF	0.762**	0.418 (0.352–0.485)	0.922**	0.358 (0.342–0.374)	0.983**	0.267 (0.252–0.282)
	TB—RF	0.730**	0.216 (0.177–0.254)	0.873**	0.091 (0.086–0.097)	0.984**	0.075 (0.069–0.080)
	GF—RF	0.942**	0.519 (0.486–0.551)	0.967**	0.256 (0.247–0.264)	0.987**	0.281 (0.272–0.290)
**Saliva 4**	TB—GF	0.805**	0.643 (0.421–0.864)	0.903**	0.228 (0.209–0.247)	0.888**	0.320 (0.293–0.347)
	TB—RF	0.507**	0.120 (0.068–0.172)	0.786**	0.046 (0.039–0.053)	0.826**	0.072 (0.064–0.080)
	GF—RF	0.847**	0.212 (0.198–0.225)	0.898**	0.216 (0.203–0.228)	0.976**	0.231 (0.225–0.236)
**Saliva 5**	TB—GF	0.679**	0.232 (0.218–0.247)	0.767**	0.161 (0.139–0.183)	0.924**	0.267 (0.244–0.290)
	TB—RF	0.635**	0.062 (0.056–0.068)	0.757**	0.027 (0.021–0.033)	0.844**	0.055 (0.048–0.062)
	GF—RF	0.948**	0.274 (0.262–0.287)	0.958**	0.190 (0.177–0.203)	0.957**	0.214 (0.206–0.222)
**Saliva 6**	TB—GF	0.118	0.041 (-0.092–0.175)	0.122	0.296 (0.048–0.545)	0.837**	0.197 (0.174–0.221)
	TB—RF	-0.013	0.013 (-0.007–0.033)	-0.016	0.042 (-0.013–0.097)	0.679**	0.038 (0.029–0.046)
	GF—RF	0.638**	0.084 (0.054–0.114)	0.933**	0.212 (0.201–0.223)	0.948**	0.220 (0.204–0.237)
**Saliva 7**	TB—GF	0.932**	0.248 (0.236–0.259)	0.887**	0.256 (0.241–0.270)	0.863**	0.262 (0.246–0.278)
	TB—RF	0.897**	0.052 (0.049–0.056)	0.848**	0.053 (0.049–0.057)	0.834**	0.053 (0.048–0.059)
	GF—RF	0.966**	0.210 (0.201–0.220)	0.967**	0.212 (0.206–0.218)	0.966**	0.211 (0.203–0.218
**Saliva 8**	TB—GF	0.985**	0.330 (0.291–0.368)	0.714**	0.286 (0.265–0.308)	0.780**	0.115 (0.098–0.133)
	TB—RF	0.933**	0.033 (0.029–0.036)	0.692**	0.071 (0.063–0.079)	0.734**	0.024 (0.016–0.031)
	GF—RF	0.928**	0.095 (0.090–0.101)	0.983**	0.259 (0.248–0.269)	0.861**	0.252 (0.216–0.287)

The Spearman rank correlations of the relative intensity of total biofilm (TB), green fluorescence (GF) and red fluorescence (RF). B is the regression coefficient of the linear regression with its 95% Confidence Interval.

** = Correlation is significant at the 0.01 level (2-tailed).

The linear character of the relationship between the different biofilm signals (total biomass, green and red fluorescence) was confirmed with scatterplots after which regression analyses were performed (Table 3). The regression analyses showed that all three methods to quantify biofilm formation were significantly associated in a linear relationship (Table 3) except for saliva six. In all cases, the regression coefficient of the linear regression was higher for total biomass versus green fluorescence compared to total biomass versus red fluorescence. Higher regression coefficients (Table 3) where found for biofilms inoculated with saliva from donors who had developed a high clinical bleeding on marginal probing score after a 14 days challenge of no oral hygiene (Table 2), when grown in the presence of serum.

## Discussion

This is the first publication describing the dynamics of green and red fluorescence of oral biofilms. Both green and red autofluorescence were found to be linearly related to the total biomass of the biofilms. All biofilms showed green and red fluorescence to some extent, with higher red and green fluorescence intensities in biofilms grown in the presence of serum as compared to biofilms grown in the presence of sucrose. The saliva donors participating in this experiment were selected on the basis of their high or low red fluorescent response to a challenge of 14 days of no oral hygiene [16]. Therefore, differences in the fluorescence response of the serum group were to be expected between these two clinically different groups when simulating gingivitis conditions (serum added). These differences were absent when simulating cariogenic conditions (sucrose added), possibly because the saliva donors were all caries free. Donor number six gave ambiguous results concerning fluorescence, indicating that individual differences in biofilm composition can be significant. Overall, red fluorescence was clearly dependent on the microbiome of the donor, since other biological characteristics of the host, like saliva, were absent in the model.

Sucrose was added in the artificial saliva medium to mimic a cariogenic environment for the biofilms [24]. The added serum represented crevicular fluid conditions to mimic subgingival plaque circumstances [25]. The higher relative red fluorescence intensities found for four of the eight inocula in the serum group were related to the inflammatory response of the saliva donors. This may indicate that when mimicking a specific clinical condition, the origin of the inoculum as well as the growth conditions for a biofilm together determine its fluorescence during early formation of biofilm. The relationship of biofilm red fluorescence with cariogenicity can be studied by inoculating the BioFlux model with saliva from caries-inactive as well as from caries-active subjects and following biofilm fluorescence under cariogenic growth conditions.

It has been previously suggested that red fluorescence, as observed from biofilms, originates from free porphyrins in the biofilm [26], though the exact origin and its clinical implications still lack a solid scientific reasoning. Even less is known about green fluorescence in biofilms. The assumption that green fluorescence is related to the total biofilm quantity is confirmed in the present study, although the extent to which it relates depends on the biofilm growth conditions as well as on differences between donors.

In clinical studies green fluorescence from plaque has never been determined. Natural teeth have the intrinsic characteristic to fluoresce green [2, 27, 28]. The red red/green fluorescence ratio used to assess red fluorescence intensity in QLF-photographs of the oral cavity or biofilms grown on dentine [13, 29], uses the green fluorescence of dentine as internal reference. The green dentine fluorescence interferes with the assessment of green fluorescence of biofilms. This may explain why green autofluorescence of oral biofilms has not been studied before, although green fluorescence from bacteria has been reported [30]. Elastin (an elastic protein in connective tissue) as well as collagen (main component of connective tissue, also present in dentin) are known to fluoresce green [31, 32]. Green fluorescence has also been reported from epidermal cells from the skin [33] as well as from healthy oral mucosal sites [34] after excitation with violet light. This green fluorescence was also seen in the epithelial cells present in the BioFlux channels at the start of the experiments as indicated by the high green fluorescence observed at the start of our experiments.

In the current study, green fluorescence as well as red fluorescence were observed during early biofilm formation, with green fluorescence in a higher intensity compared to red fluorescence. This can partially be attributed to differences in response of the imaging system. The typical spectral response of a black and white CCD chip is a Gaussian curve with the highest sensitivity in the green part of the visible spectrum and low sensitivities towards the ultraviolet and infrared part of the spectrum. We partly compensated for this by selecting an emission filter for the red part of the spectrum with a large bandwidth. Despite this, the red fluorescence signal was much lower than the green fluorescence signal and a longer exposure time was needed to observe the signal. Hence, we assume that the green fluorescence signal from young biofilms is indeed higher than the red fluorescence signal. This would be in agreement with our previous study reporting green biofilm fluorescence after a few days and red fluorescence taking over in time [15]. Both green and red fluorescence seems to be related to total biomass, though to a different extent.

Interestingly, both the red and green fluorescence are location specific and not homogeneously distributed within the biofilms, while being observed on the same locations in the biofilm. Unfortunately, the model used in the present study does not allow for sampling to study the composition of these high fluorescent spots. It can be of future interest to determine if highly fluorescent spots relate to a microbiome (hence metabolome) associated with health or disease.

Red fluorescence has been reported from bacteria related to periodontal inflammation as well as from bacteria related to caries [12, 30, 35–37], while green fluorescence from bacteria has also been reported [30]. A drawback from these studies is that they did not assess the characteristics of the interactions between oral bacteria. Recent research focused on fluorescence from microcosm biofilms inoculated with saliva [13, 15, 29] as this mimics the clinical situation with dental plaque on the tooth surface. In these previous studies biofilms were grown under static conditions. In the BioFlux model, biofilms are grown under flow conditions mimicking the clinical situation even better. The BioFlux is limited in biofilm build-up due to the small dimensions of the channels, which are vulnerable to clogging. Therefore, it was not useful to continue the current experiments beyond the presented 12 hours. Also, the three-dimensional nature of biofilms would require a three-dimensional analysis, e.g., using confocal microscopy.

The remarkable chain length of cocci in the biofilms, presumably streptococci, has not been described before in detail. However, it is known that chain lengths are variable [38]. The presence of antibodies can influence the chain length of streptococci [39, 40]. It has been reported that small chains of *Streptococcus mutans* in monocultures are more sensitive to an aciduric stress [41]. It has also been reported that the chain length of streptococci is dependent on the growth conditions and consequently on the growth rate [42]. Therefore, it is possible that the long chain length is the result of the abundant and continuous availability of growth substrates and continuous removal of growth-inhibiting waste products in our model, enabling logarithmic growth and inhibition of active de-chaining processes. Future research may include the effect of medium flow and biofilm aging on the chain length of these bacteria and the effect on the virulence of a biofilm.

In dentistry, there is a growing interest in developing non-invasive, easy to use and inexpensive methods for the early detection of patients who are at high risk for developing oral diseases. A reliable biofilm model to mimic the characteristics of fluorescence from oral biofilm is the first step to understanding fluorescence from dental plaque and estimating its potential to use these characteristics for risk assessment of patients. The BioFlux equipment proved to be a flow model suitable to assess biofilm fluorescence in time. Green and red fluorescence from biofilm was seen very early in biofilm formation. With this study, we contribute new insights to the previously found association between red fluorescence and gingival inflammation. The red fluorescence increase in the grown biofilms appeared to be related to the inflammatory response of the respective saliva donors, as assessed clinically by bleeding on marginal probing after a period of 14 days of no-oral hygiene. With mimicking these results *in vitro*, red biofilm fluorescence proves to be related to the oral microbiome and not (only) with the biological characteristics of the host. Consequently, red fluorescence seems to have potential to identify a dysbiotic oral flora and thereby the presence of gingivitis related oral biofilms.

## Supporting Information

S1 Table Excel-file with the raw data from all experiments. (XLSX)

S1 Fig Graphs of the average biofilm growth with its fluorescence from all eight inocula and all three growth conditions. The graphs show the relative percentage of biofilm growth with its green and red fluorescence.
(TIFF)

S1 Movie Typical example of a time-lapse movie of biofilm formation with green fluorescence (serum, anaerobe). (AVI)

S2 Movie Typical example of a time-lapse movie of biofilm formation with red fluorescence (serum, anaerobe). (AVI)

S3 Movie Typical example of a time-lapse movie of biofilm formation with green fluorescence (sucrose, anaerobe). (AVI)

S4 Movie Typical example of a time-lapse movie of biofilm formation with red fluorescence (sucrose, anaerobe). (AVI)

S5 Movie Typical example of a time-lapse movie of biofilm formation with green fluorescence (sucrose, aerobe). (AVI)

S6 Movie Typical example of a time-lapse movie of biofilm formation with red fluorescence (sucrose, aerobe). (AVI)
