# Supplementary figures and images for: Red and Green Fluorescence from Oral Biofilms
Source: PLoS One. 2016 Dec 20;11(12):e0168428. doi: 10.1371/journal.pone.0168428 (PMC5173178; doi:10.1371/journal.pone.0168428)

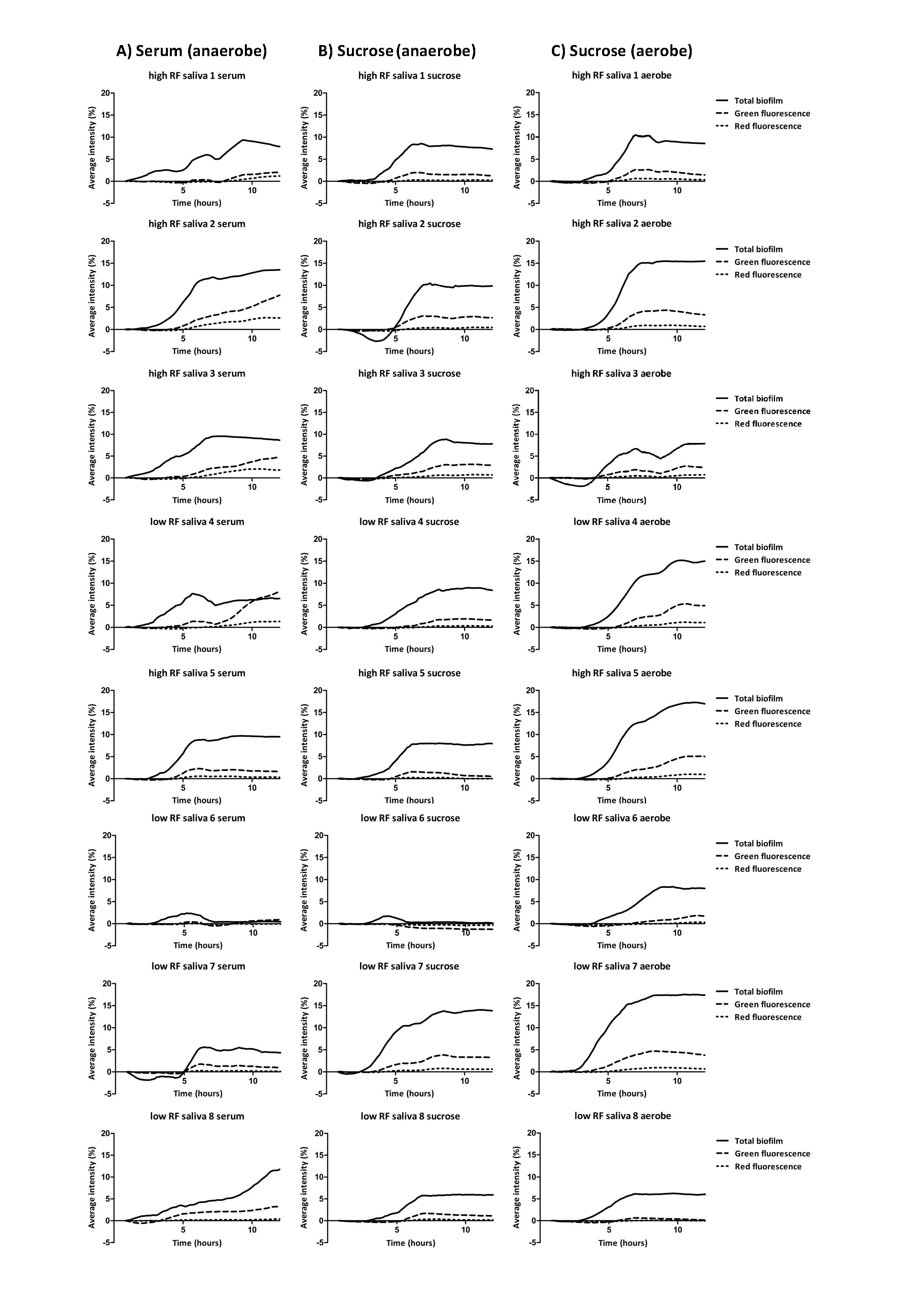

Supplement: S1 Fig — The graphs show the relative percentage of biofilm growth with its green and red fluorescence. (TIFF) [file pone.0168428.s002.tiff]
